# A Survey of Transoral Robotic Mechanisms: Distal Dexterity, Variable Stiffness, and Triangulation

**DOI:** 10.34133/cbsystems.0007

**Published:** 2023-03-13

**Authors:** Xiaoyi Gu, Hongliang Ren

**Affiliations:** ^1^ Department of Electronic Engineering, The Chinese University of Hong Kong, Hong Kong, China.; ^2^ Suzhou ACTORS Medtech Co., Ltd, Suzhou, Jiangsu, China.

## Abstract

Robot-assisted technologies are being investigated to overcome the limitations of the current solutions for transoral surgeries, which suffer from constrained insertion ports, lengthy and indirect passageways, and narrow anatomical structures. This paper reviews distal dexterity mechanisms, variable stiffness mechanisms, and triangulation mechanisms, which are closely related to the specific technical challenges of transoral robotic surgery (TORS). According to the structure features in moving and orienting end effectors, the distal dexterity designs can be classified into 4 categories: serial mechanism, continuum mechanism, parallel mechanism, and hybrid mechanism. To ensure adequate adaptability, conformability, and safety, surgical robots must have high flexibility, which can be achieved by varying the stiffness. Variable stiffness (VS) mechanisms based on their working principles in TORS include phase-transition-based VS mechanism, jamming-based VS mechanism, and structure-based VS mechanism. Triangulations aim to obtain enough workspace and create adequate traction and counter traction for various operations, including visualization, retraction, dissection, and suturing, with independently controllable manipulators. The merits and demerits of these designs are discussed to provide a reference for developing new surgical robotic systems (SRSs) capable of overcoming the limitations of existing systems and addressing challenges imposed by TORS procedures.

## Introduction

### Clinical significance

Head and neck cancers (HNCs) include a group of malignant tumors that primarily develop in or around the upper aerodigestive tract (UAT) [1,2]. More than 710,000 new cases and 360,000 deaths are reported with HNCs annually worldwide [3], which is still increasing in incidence [1,3–6]. The face and neck are important for human identity and appearance. Surgery is a frequently employed and essential treatment for most types of HNCs, intending to remove cancerous cells [7,8]. In a traditional head and neck surgery, to obtain adequate exposure to the primary lesion and the surrounding tissues/organs, the lip, mandible, and neck of a patient have to be incised or divided [8,9], which inevitably causes serious traumas, fatal complications, cosmetic deformities, and functional impairments to the patients [1].

### Minimally invasive transoral surgery

Surgical treatment paradigms for HNCs include (a) traditional open surgery [10], (b) minimally invasive transoral surgery [11], and (c) transoral robotic surgery (TORS) [12].

Minimally invasive surgery (MIS) targeted at reducing invasiveness resulted from traditional open approaches, which realized the technical revolution when distal cameras were available [13]. With digital cameras, surgeons can have a better view of the surgical site via monitors in real time instead of peering through the endoscope lens, which frees their hands to manipulate surgical instruments. In an MIS procedure, multiple instruments together with endoscopes are inserted through several small incisions or natural orifices to reach surgical sites that were previously only accessible through open approaches [14,15]. Compared with the traditional open approaches, MIS can benefit patients with less blood loss, reduced morbidity, decreased pain, shortened recovery time, and improved patient outcomes. It is also cosmetically beneficial because only small or no scars are left on patients [16]. With the advancement of technology, MIS techniques are now being applied in more surgical procedures [17].

As a typical MIS procedure, transoral surgery (TOS) was initially coined for procedure related to oropharyngeal and laryngeal cancers [18] and is now being expanded to broader applying scopes [19–25]. The mouth is taken as the entry port in a TOS procedure, and the laryngoscope provides the working channels [26]. In addition to retaining the advantages of MIS, TOS can eliminate incision-related complications, minimize functional impairment to the UAT, and achieve improved postoperative outcomes [27]. However, this MIS technique is facing the bottleneck of development owing to long and stiff surgical instruments, which are limited to a few degrees of freedom (DOFs) because of the constraint of the insertion port and lack of enough dexterity at the distal end. Additionally, the insertion port acts as a pivot point on the instruments, and the variable motion scaling is determined by the insertion depth [28,29]. Thus, surgeons shall overcome a steep learning curve before grasping these technologies. Moreover, manual laryngeal surgery must be performed with a suspension laryngoscope, which has a high risk of inducing various complications [30,31].

### Robotic solution to TOS

The demand for robotics is increasing for less invasive surgical technologies in the realm of medicine [15,32–35], which is not to replace the surgeons completely but to supplement or extend their abilities to obtain better treatment outcomes. Therefore, surgical robots are considered surgeon extenders [32,36]. Early surgical robots, e.g., Unimation PUMA 200 [37], ROBODOC [38], and PROBOT [39], are the reconstruction of conventional industrial robots with modifications for safety and sterility [40], which are only applicable for basic stereo positioning. AESOP, as the Food and Drug Administration (FDA)-approved laparoscopic camera holder [41], greatly expanded the concept of robotic surgery to date and paved the way for the development of endoscopic robots. ZEUS is a robotic surgical system (Computer Motion, Inc., Goleta, CA, USA) that consists of an AESOP arm to maneuver the endoscope and 2 arms fitted with interchangeable instruments for surgical operations, which received FDA approval for limited procedures in 1996 [42]. The da Vinci surgical robotic system (SRS) (Intuitive Surgical Inc., Goleta, CA, USA) [33] comprises a master console, a patient cart, and an imaging system. After getting FDA approval in 2000, da Vinci systems have continuously evolved to fit an extensive range of applications [43]. In 2019, da Vinci systems performed approximately 1,229,000 surgical procedures, which increased by 18% compared with the data in 2018 [44]. Smaller, smarter, and safer surgical robotic platforms are being developed and targeted at broader clinical applications [33,45].

The application of surgical robots in TOS is referred to as TORS. A TORS procedure was completed in animal models with the da Vinci system in 2003 [46]. Two years later, TORS was successfully performed on a human subject using the same system [47] and then obtained FDA approval for oropharyngeal cancer in 2009. Since then, the clinical application of TORS in the management of HNCs has expanded considerably [48]. Recently, a novel single-port SRS (da Vinci single port [SP]) [49] developed by Intuitive Surgical Inc. is commercially available and applied in TORS, the technical feasibility of which is safe access to deep surgical sites of the UAT that was verified by early results of clinical trials [50,51]. Flex SRS (Medrobotics Inc., Raynham, Massachusetts, USA) is another system that received clearance from the FDA for TORS in 2015, which has been successfully adopted in the management of oropharyngeal tumors [50,52].

Robotic technologies are integrated into MIS procedures to overcome drawbacks caused by rigid surgical tools and their associated fulcrum effects, which are capable of benefiting patients with reduced trauma, shortened hospital time, increased diagnostic accuracy, and improved therapeutic outcomes while alleviating cognitive and physiological loading of surgeons [15,32–35]. The majority of existing SRSs adopt dexterous wrists to allow surgeons to complete complex tissue manipulations that are very difficult to achieve with manual surgical tools, which contributes to the great success of robot-assisted MIS in the extraluminal procedure that has a shallow and wide surgical area. Nevertheless, these devices encounter difficulties in the intraluminal procedure, characterized by deep and confined surgical sites. To extend the ability and application scope of the surgical robot in MIS, specialized designs have been or are being developed in the past 3 decades [33–35,45,53,54].

### Advantages of TORS

TORS has completely revolutionized the diagnosis and treatment of diseases in the UAT, the advantages of which include:

• The master-slave control mode employed by the SRS is effective in removing hand tremors and mitigating the side effects of fatigue and inattention caused by prolonged operation [32,33]. Moreover, the master console can be located away from the patient to protect surgeons from infection and radiation.
• Target surgical sites in the UAT can be directly visualized and reached through the mouth, which avoids the mandibulotomy and rigid laryngoscopes to reduce the volume of disrupted tissues and alleviate function impartment [18]. Furthermore, this direct approach can eliminate the scar on the patient’s body and reduce postoperation complications, including wound site infection, hernia, and adhesion [15].
• Robotic instruments have more controllable DOFs at the distal end and improved hand-eye coordination, which can restore and even augment surgeons' reduced motor skills in confined surgical areas [28,55,56].
• In TORS, the reduced possibility of edema in the UAT leads to a decreased risk of postoperative airway obstruction. Thus, a permanent tracheostomy is not required, and the temporary tracheostomy can be avoided in many cases [18,57,58].
• The time required by patients that had TORS to return to basic swallowing function, as well as the average hospital time of these patients, can be considerably reduced, which can effectively lighten the economic weight of patients [57,59–61].
• In addition to the faster postoperative recovery and increased functional outcomes, the oncological outcomes of TORS can also be remarkably improved in terms of the survival rate owing to the accurate and timely stage of HNCs as well as the more efficient and precise surgical operations [9,57,59].

As aforementioned, there have been several SRSs adopted in TORS. Although they have achieved some encouraging outcomes and been approved for marketing, they cannot address most of the specific technical challenges presented by TORS. Multiarmed da Vinci SRSs are the most widely adopted in TORS. The interference between their bulky, rigid, and slender working/camera arms or mouth retractors is inevitable [62], challenging the access of deep sites in the UAT. Therefore, these systems only apply to selected malignant lesions or benign diseases in the oropharynx and larynx [63]. The newly launched single-port da Vinci surgical system ameliorates the problem of interference and has a relatively compact structure to reduce restrictions presented by individual patient anatomy. Although the technical feasibility of this system is verified [51], no evidence indicates that it can cover the whole target area. Besides, the long-term functional and oncological outcomes remain to be investigated by more in-human trials. The Flex robotic system is categorized as the next-generation flexible robot, which has a snake-like structure that can provide enough DOFs and better flexibility for the robot to access deep anatomical sites via confined and nonlinear pathways [50,64]. Nevertheless, broad cross-section, slow running speed, small load capacity, and manually controlled surgical instruments lead to lower levels of accuracy and manipulability in TORS.

### Challenges

TORS is classified into intraluminal surgery and is distinct from the current commonly adopted extraluminal procedures [35]. The taxonomy and specific technical challenges of these 2 types of MIS procedures are presented in Table 1. Before an extraluminal procedure, the gas would be insufflated into potential anatomical space to create a large space for surgical operations. In addition, the surgical sites are usually near the incision ports. As a result, the extraluminal procedure features shallow and wide surgical fields. There are many technical challenges of the extraluminal procedure, including the fulcrum point effect and the insufficient distal dexterity of the tool caused by the incision constraint, interference between multiple instruments, impaired hand-eye coordination, and obstructed field of view. Most of them have been handled by current robotic technologies, which adopt multiarmed or single-port designs and incorporate dexterous wrists. Although lack of force feedback brings difficulties to some dexterous and high-precision operations, depth perception provided by the 3-dimensional (3D) stereo camera can make up for this deficiency to a certain extent [15,35]. Consequently, robot-assisted extraluminal procedures, e.g., laparoscopy and thoracoscopy, represent the vast majority of current robotic surgeries [35].

**Table 1.** Taxonomy and specific technical challenges of the extraluminal and intraluminal procedures

	Extraluminal procedure	Intraluminal procedure
Typical configuration	Surgical instruments are inserted through multiports or an SP in the patient’s body to access the target surgical site.	Surgical instruments access the target surgical site via natural orifices of patients and complete surgical operations within an anatomical lumen.
Challenges	• Fulcrum point effect • Insufficient tool dexterity • Multiple instruments • Impaired hand–eye coordination • Obstructed visualization • Sensory deficiency	• Insertion ports with various size • Indirect, long, and tortuous access routes • Contact with multiple points • Dexterity and maneuverability in deep and narrow workspaces

As a typical intraluminal procedure, TORS takes the mouth as the insertion port to further reduce the scarring and postoperative complications characterized by deep and narrow operative workspaces [15]. In addition to the challenges above, TORS impose higher demands for developing robotic surgical systems owing to the complex anatomical structure of the UAT. The oral cavity size is markedly different between individuals, which requires the robot to have a smaller size to alleviate this impact. Unlike in the extraluminal procedure, where only the distal part of the robot would contact the anatomy, surgical robots for the intraluminal procedure should have more DOFs and larger workspaces to traverse in-direct, long, and tortuous intrabody passageways. Additionally, these robots should have adequate flexibility and conform to the anatomy to protect surrounding tissues from contact with multiple points or regions. However, increased flexibility always leads to the decreased load capacity of the robot, the contradiction between which remains a serious problem in the design of surgical robots for TORS.

Moreover, multiple robotic instruments with adequate distal dexterity are essential in deep and narrow surgical procedures. Their shafts have to converge through the same confined access path. Coordinating these robotic instruments to obtain a suitable working configuration and ensure enough maneuverability for surgeons presents a great challenge for the robot design.

## Distal Dexterity Mechanism

The distal dexterity of a surgical robot refers to the capability of robots to move and orient end effectors [65], which plays a vital role in restoring and even augmenting the motor skills of surgeons in the surgical field and has been the core research topic since the advent of the surgical robot. According to their structure features, these designs can be classified into 4 categories: serial mechanism, continuum mechanism, parallel mechanism (PM), and hybrid mechanism.

### Serial mechanism

**Fig. 1.**
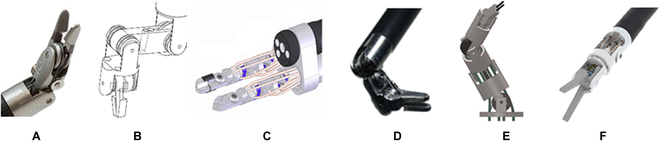
Surgical robots based on pin joint mechanisms. (A) EndoWrist Instrument® [166]. (B) The Black Falcon [167]. (C) EndoMaster [168]. (D) A handheld mechatronic laparoscope surgical robot [169]. (E) A magnetic resonance imaging-guided SMA spring-actuated neurosurgical robot [70]. (F) MicroHand [170].

The serial mechanism has 2 obvious features: (a) Joints are limited and discrete, and (b) each joint can be independently controlled. The most typical design is the cable-driven pin joint mechanism, which has been widely employed in the design of the surgical robot, as shown in Fig. 1. Each pin joint can be independently controlled by a pair of cables that can transfer actuation force through long and nonlinear routes to allow actuators to be separated from the robot body, which contributes to the compact structure of the robot. However, the linkages of these robots are rigid, and cables are always under tension to reduce the extension/slack, which leads to the high stiffness of these robots. Moreover, interference between joints and some undesired nonlinear characteristics due to the stretching and friction of cables leads to the degradation of the accuracy of the robot [53,66]. Although some compensation methods, including modified joint mechanisms [67–69] and sophisticated control algorithms [70,71], have been proposed to improve the accuracy of the robot, the influence caused by the coupling motion of joints and nonlinearities of cables cannot be eliminated.

**Fig. 2.**
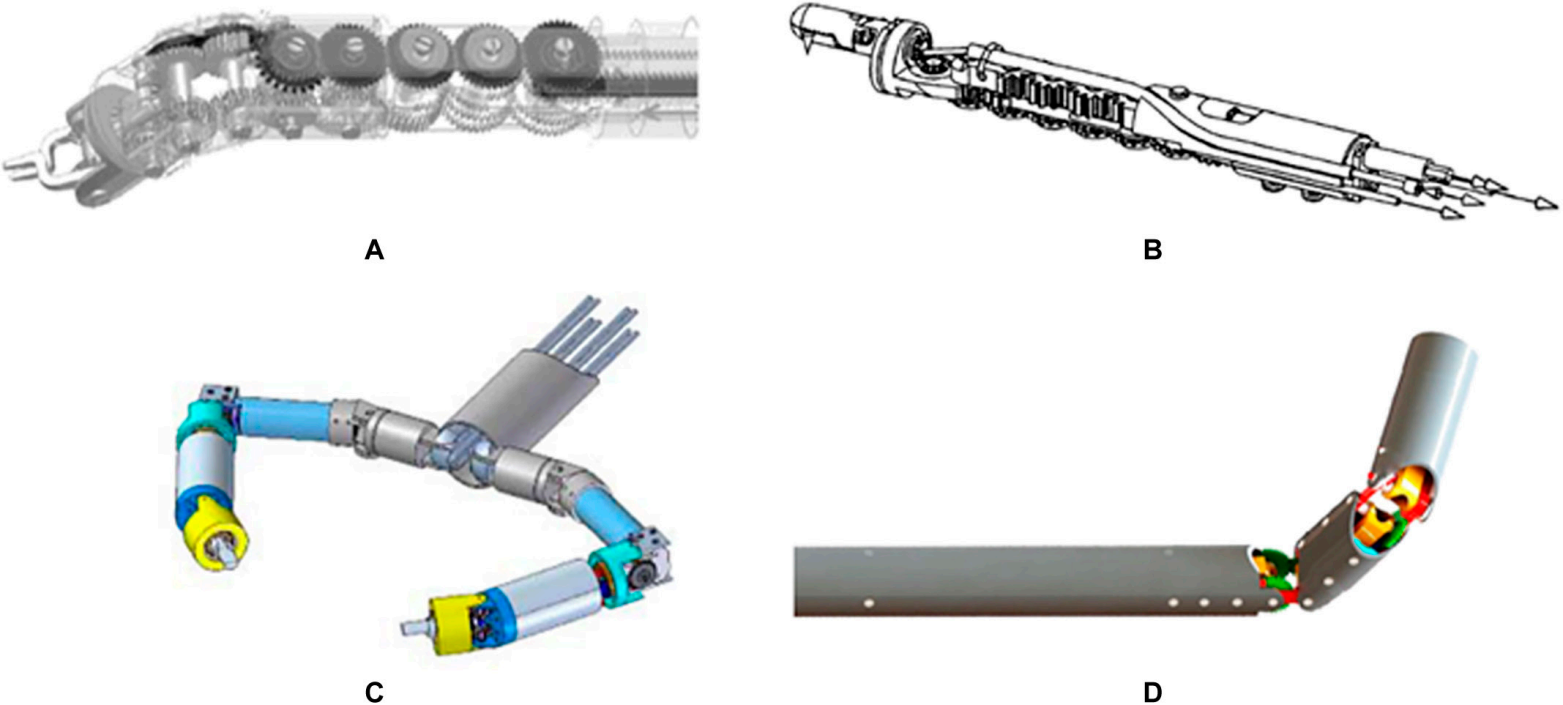
Surgical robots based on gear-driven mechanisms. (A) A multi-DOF forceps manipulator [171]. (B) Articulated manipulator for minimally invasive surgery [71]. (C) SPRINT [172]. (D) Multigear array mechanism [173].

A gear-driven mechanism is characterized by high stability, durability, efficiency, and long life span, which has been employed to enhance the distal dexterity of the surgical robot (see Fig. 2). However, these robots have extremely high stiffness and can be considered stiff. Additionally, backlash caused by clearance allowances and assembly errors negatively affects the accuracy and repeatability of the robot. Improving the accuracy of gears can result in a rapid increase in manufacturing costs.

**Fig. 3.**
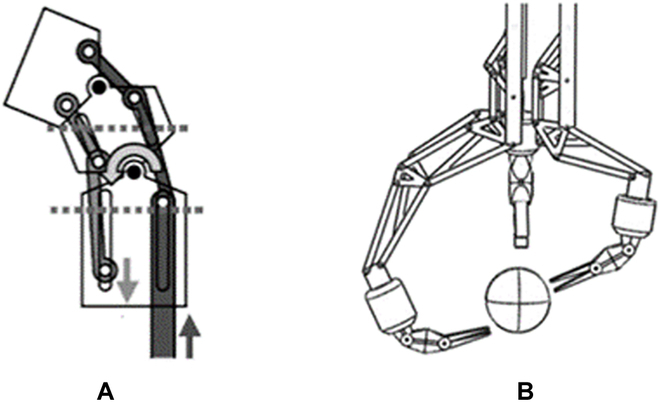
Linkage-based mechanisms. (A) Multislider-linkage mechanism [174]. (B) Stackable 4-bar mechanism [74].

Recently, several linkage-based distal dexterity mechanisms have been proposed, as illustrated in Fig. 3. Figure 3A plots the multislider-linkage mechanism, which has been verified to have the ability to generate a large workspace and reduce the hysteresis caused by the backlash to enable high accuracy [72,73]. Nevertheless, the dexterity is difficult to enhance further because additional DOFs would dramatically increase the structure's complexity. A stackable 4-bar mechanism is another delicate design that is adopted to achieve dexterous distal motions (see Fig. 3B), which has the merits of lightweight and slim size [74]. Despite these advantages, the dexterity of this mechanism is restricted to 2-dimensional planar motion.

In summary, serial mechanisms usually utilize limited, rigid links and stiff joints to achieve desired motions. Since all joints are independently controllable, modeling these robots is relatively simple. However, the incapacity of both stiff materials and joints leads to the high stiffness of the robot. Although the high stiffness contributes to the enhanced load capacity, it is an undesired characteristic from the view of safety, as safety is one of the main considerations that decide the usage of different mechanisms in the medical field. Consequently, careful motion planning is necessary to avoid contact between the robot and the surrounding human tissues.

Nevertheless, difficulties in accurately reconstructing the target anatomy and nonlinear pathways bring much trouble to motion planning. Moreover, the dexterity of a serial robot is determined by the number of DOFs, the increase of which leads to either more complex structures or larger dimensions of the robot. The size and complexity issues can deteriorate if independent motors are needed for each independent joint of the structure.

### Parallel mechanism

**Fig. 4.**
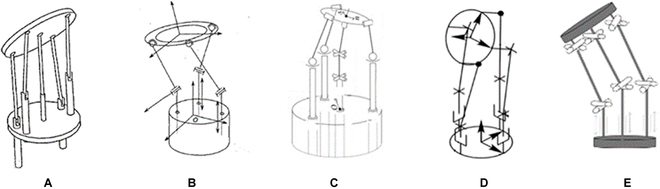
Schematic diagrams of the PMs that are employed in the development of surgical robots. (A) 4-PUS with a central pivot [75]. (B) 3-PRS [76]. (C) 3-PRS with an additional revolute-universal-universal-prismatic leg [77]. (D) 2-PUU-2-PUS [78]. (E) 3-PUU [79].

**Fig. 5.**
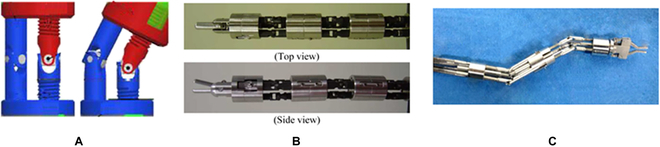
DSD mechanism. (A) Side view of the mechanism [80]. (B) A DSD forceps manipulator [80]. (C) A pediatric surgical robot based on the DSD mechanism [81].

PM, which features high precision, load capacity, and reliability, has also been employed to enhance the distal dexterity of MIS surgical robots. A variety of configurations have been proposed, as illustrated in Fig. 4, including a 4-PUS (prismatic-universal-spherical) PM with a central pivot [75], and optimized 3-PRS (prismatic-revolute-spherical) PM [76], a 3-PRS PM with an additional revolute-universal-universal-prismatic leg [77], a 2-PUU (prismatic-universal-universal)-2-PUS PM [78], and 3-PUU PM [79]. Besides, the screw drive mechanism is integrated into the PM to form a novel bending mechanism termed the double screw drive (DSD) mechanism (see Fig. 5A), which can generate universal bending motions by rotating the linkages with threads at both sides [80]. Using the DSD mechanism, a dexterous forceps manipulator [80] and a pediatric surgical robot have been developed [81] (see Fig. 5B and C).

In addition to the inherent advantages, robots designed on the basis of PMs feature better dexterity because the joint formed by the PM can generate bending motions in all directions with a smaller bending radius, which is as dexterous as the human wrist. Nevertheless, robots with these structures are regarded as rigid devices with poor adaptability because of the extremely high stiffness, an undesired characteristic of MIS. Additionally, it is not trivial to obtain closed-form kinematics solutions for most parallel robots [82]. Moreover, parallel robots have a high requirement for assembly accuracy. Scaling down the size of an MIS parallel robot imposes a great challenge for the manufacturing capability because it is extremely difficult to produce spherical hinges or revolute hinges of small size while ensuring accuracy and reliability.

**Fig. 6.**
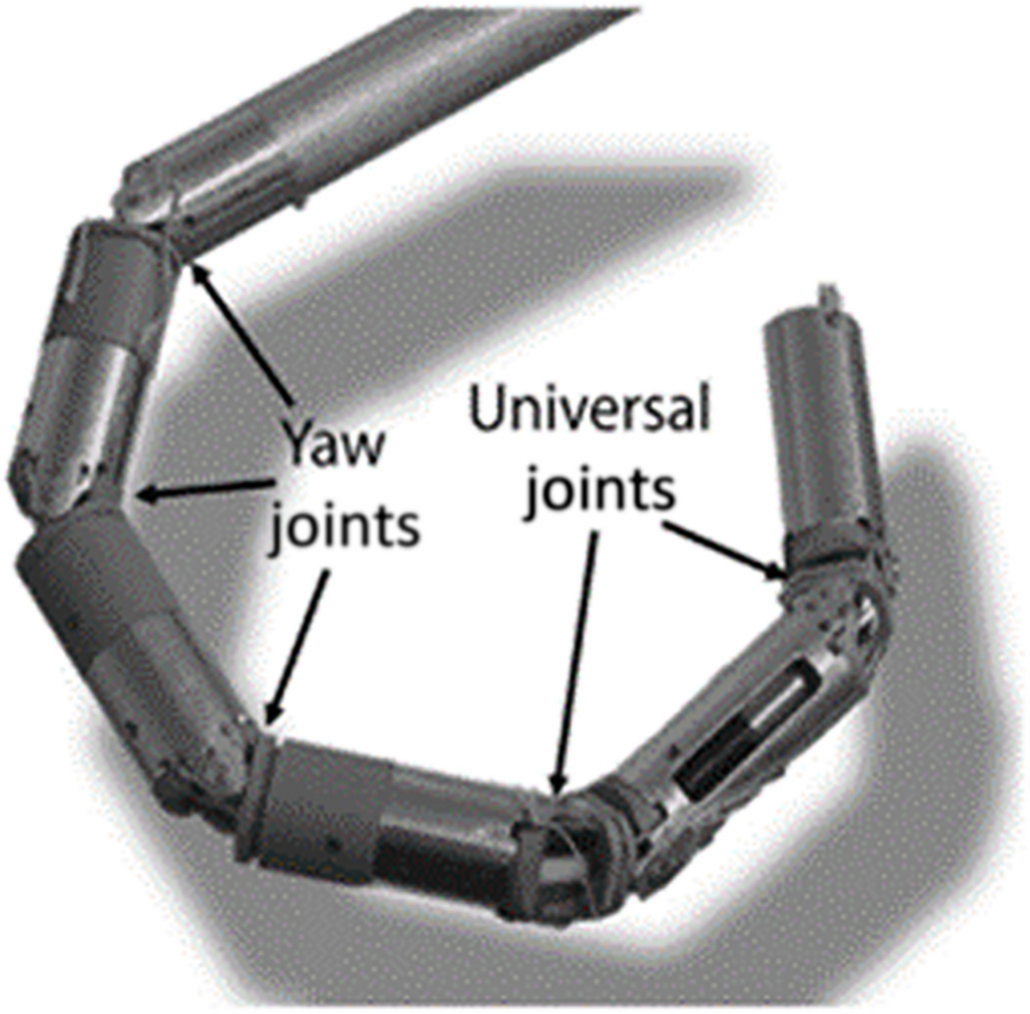
“i-snake” surgical robot [83].

Hyper serial structures employ a number of discrete rigid links. Figure 6 illustrates the mechanical structure of the “i-snake” surgical robot, composed of a few independently controlled universal joints and yaw joints [83]. This fully controlled mechanism achieves improved distal dexterity at the cost of complex structure and control algorithms, which brings great difficulty in minimizing the robot size. Moreover, the load capacity of this robot after minimization is very limited [84].

**Fig. 7.**
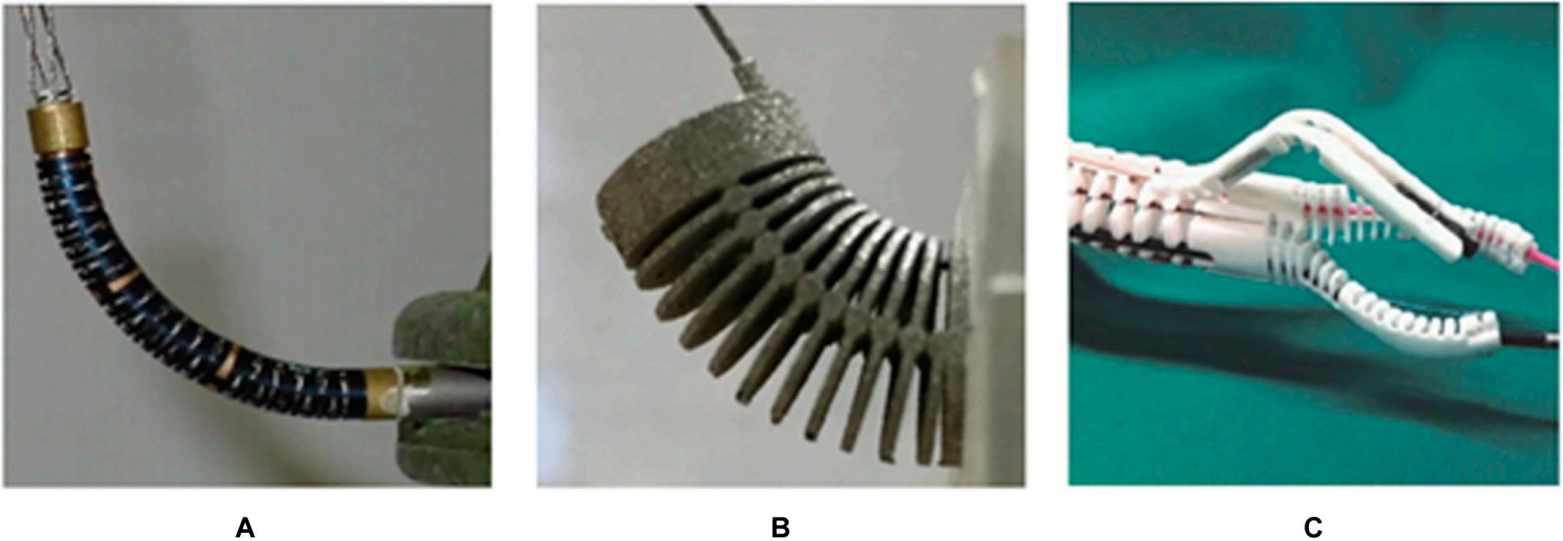
Continuum mechanisms with elastic structures are produced by manufacturing techniques, including (A) laser cutting [90], (B) metal 3D printing [91], and (C) selective laser sintering [92].

### Continuum mechanism

Previous papers and review articles have provided a strict definition of the continuum mechanism, which is inclusive of some specific descriptions, such as “infinite DOFs”, “continuum bending”, “elastic structure”, and “do not contain any rigid links or discrete joints” [85–87]. As a result, most continuum mechanisms adopt the elastic structures and tendon-driven under-actuation approaches to achieve the bending motion, which can greatly simplify the structure and reduce the robot size.

There are approaches to building the elastic structure of the continuum mechanism, as illustrated in Fig. 7. The most direct method is to produce grooves on elastic tubes using high-precision laser cutting machines [88–90]. In addition, some advanced additive manufacturing techniques, e.g., metal 3D printing [91] and selective laser sintering [92], have also been applied to fabricate the elastic structures. Although these mechanisms offer simple structures and reduced size, they suffer from high manufacturing costs and a large bending radius.

**Fig. 8.**
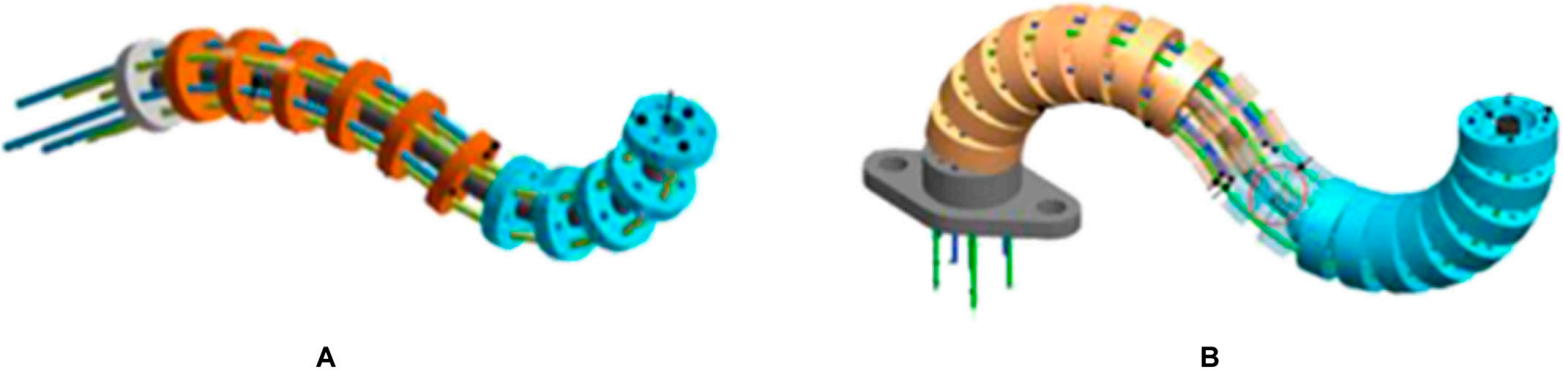
Commonly used configurations in the design of the continuum mechanism [28,93–96]. (A) Tendon-driven single- or multiple-backbone continuum mechanisms. (B) Tendon-driven serpentine mechanism.

Figure 8 shows the 2 commonly used configurations in the design of the continuum mechanism. As shown in Fig. 8A, a number of spacer disks containing some circumferentially and equidistantly distributed pinholes to guide tendons are fixed to single or multiple elastic backbones to form the elastic structure of the continuum robot [28,93–96]. Similar to this configuration, the continuum mechanism illustrated in Fig. 8B comprises sequentially linked vertebrae and an elastic beam, termed the serpentine mechanism for distinction. Two successive vertebrae can form either a revolute joint [97–100] or a spherical joint [101–105], the bending motion of which can be constrained and regulated by the elastic beam. These 2 continuum mechanisms have similar characteristics, including simple structures, easy construction, small size, and low stiffness. Nevertheless, their workspace and dexterity are reduced compared with fully actuated serial robots.

**Fig. 9.**
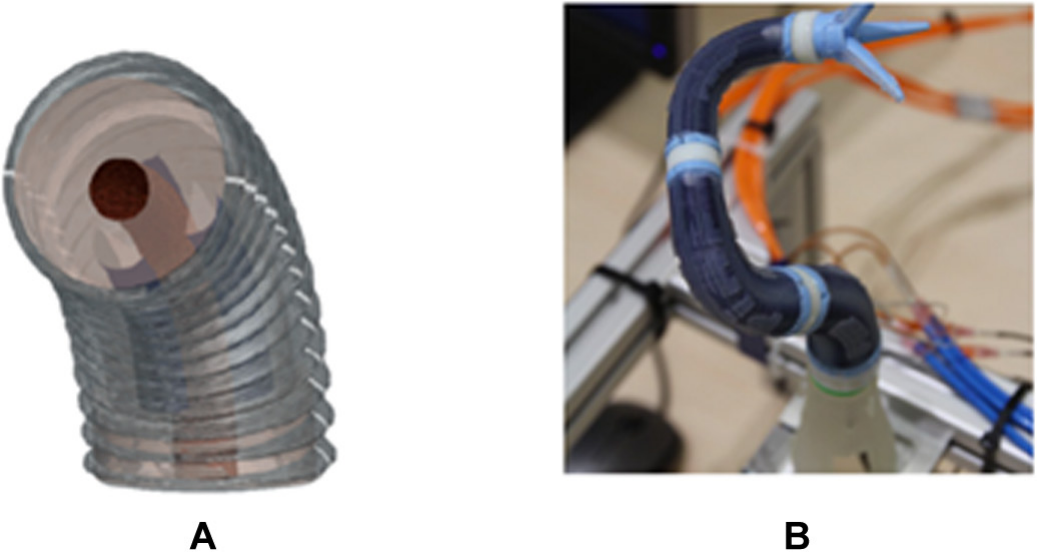
Pneumatic soft mechanism. (A) Single module [175]. (B) Stiffness Controllable Flexible & Learnable Manipulator for Surgical Operations (STIFF-FLOP) [149].

A soft pneumatic mechanism made from silicon materials can also be considered a continuum mechanism. Each module can achieve 2-DOF bending and 1-DOF translational motion by adjusting the air pressure of the air chambers (see Fig. 9A). The workspace and distal dexterity of the soft pneumatic robot can be significantly increased by stacking multiple modules together, as shown in Fig. 9B. Although this mechanism has high flexibility and compliance to ensure adequate safety, low control accuracy and limited load capacity hinder its application in the medical field.

**Fig. 10.**
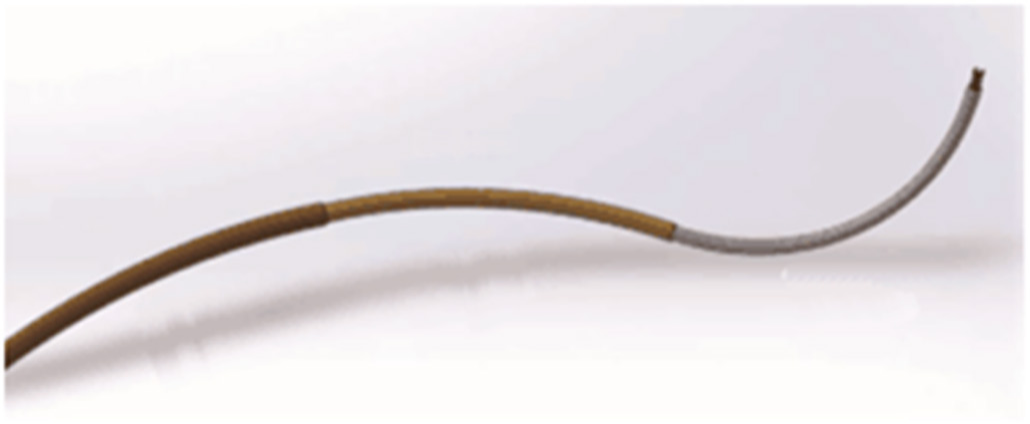
Concentric tube mechanism [112].

Different from the above designs, the concentric tube mechanism (CTM) (see Fig. 10) contains several nested precurved super elastic tubes [106–111]. The deformation and orientation of each tube are determined by the rotational angle and the extended length of the bending section. For 1 CTM, the final shape of the backbone is the resultant shape of multiple sections with different curvatures. It is possible to scale down the size of a CTM to submillimeters [111]. However, the workspace and dexterity of this mechanism are limited because the curvatures of super elastic tubes are predefined, making the CTM unable to access some target areas or unable to fulfill some required tasks with specific poses. The dexterity of the concentric tube robot is weaker than that of other continuum robots, only when the number of segments is the same. Furthermore, material fatigue is another problem that remains unsolved. CTM can enhance the reachable working space and dexterity by incorporating other tendon-driven hyper-redundant continuum mechanisms.

Compared with other mechanisms, the continuum mechanism is easier to minimize and has smaller stiffness to achieve better flexibility. Additionally, its intrinsic compliance can remarkably improve the adaptability of surgical robots in confined working environments. As a consequence, they are widely adopted in the field of surgical robots. However, reduced stiffness leads to decreased load capacity and lower motion accuracy. Besides, the modeling analysis of the continuum mechanism is usually more difficult than other mechanisms. Sophisticated numerical methods, such as the iterative Newton-Raphson method or the Levenberg-Marquardt method [112], are required to obtain the numerical solution of its inverse kinematics. Although the workspace and distal dexterity of the continuum mechanism can be extended by segmenting the backbone to multiple bending sections by tendon knots or stacking more mechanisms together, these methods will considerably increase the modeling complexity and reduce the stability of the robotic system.

### Hybrid mechanisms

Hybrid mechanisms emerge when a single mechanism has respective pros and cons and cannot satisfy all the clinical application requirements.

**Fig. 11.**
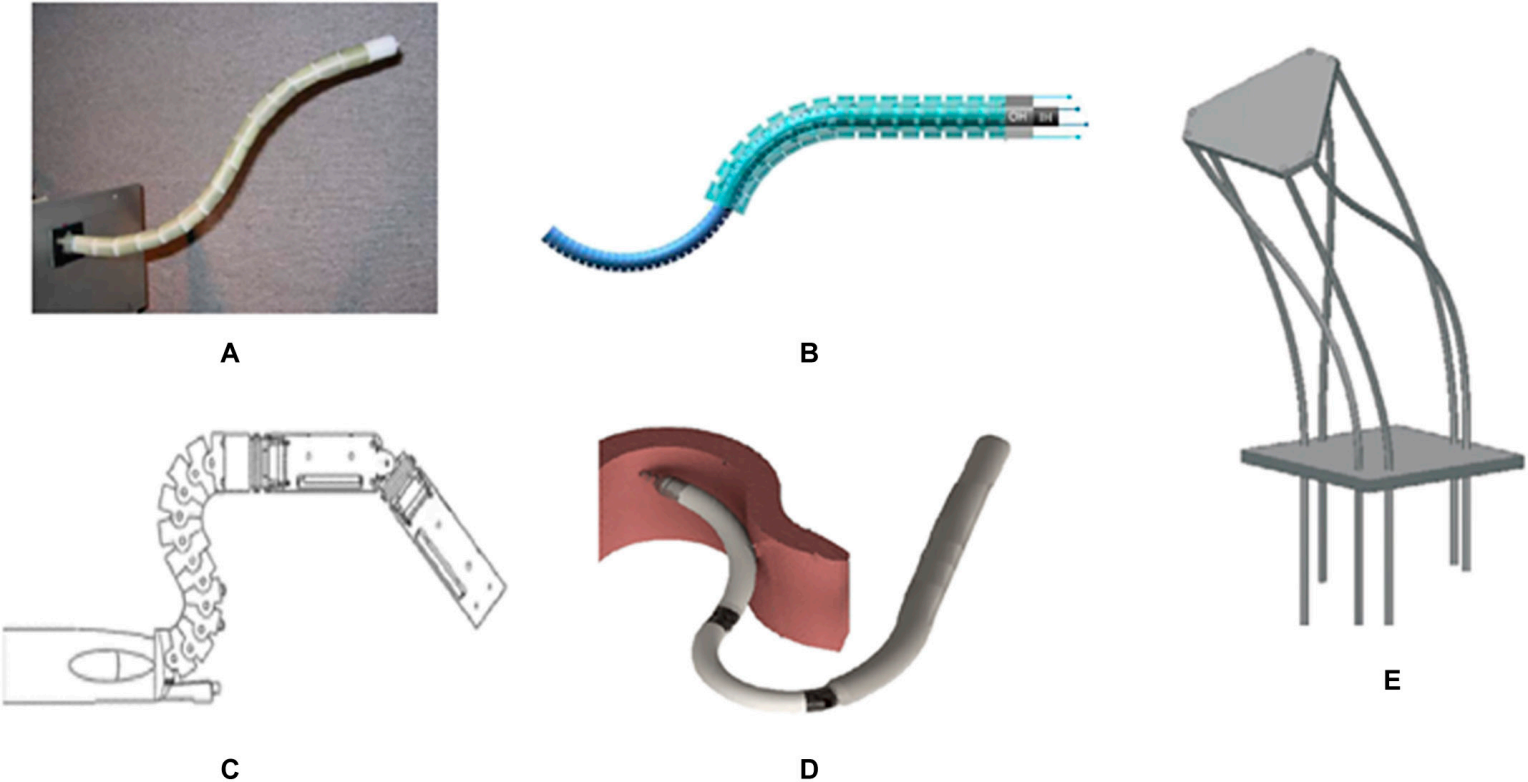
Surgical robot based on hybrid mechanisms. (A) HARP [113]. (B) Concentric wire-driven mechanism [114]. (C) A multitasking robotic platform [115]. (D) Interleaved continuum-rigid manipulator [176]. (E) Parallel continuum mechanism [116].

As shown in Fig. 11A and B, the tendon-driven serpentine mechanism and the CTM design concept are combined in developing the surgical robot. In the design of the highly articulated robotic probe (HARP) robot [113], 2 serpentine mechanisms were concentric and could alternate between being rigid and limp. The rigid continuum mechanism could work as the constraint and guide the limp mechanism to advance 1 link. The HARP robot could follow an arbitrary 3D curve by continually alternating the rigidity and limpness of the outer and inner robots. The concentric wire-driven mechanism adopts a similar structure [114]. Two continuum mechanisms with different stiffness were nested. The outer continuum mechanism, which has relatively high stiffness, serves as the active constraint to control the length and angulation of the bending section of the inner continuum mechanism. Both simulation and experimental results illustrated that surgical robots' flexibility, dexterity, and workspace with this type of hybrid mechanism were substantially expanded.

Figure 11C and D illustrates surgical robots developed using a hybrid mechanism that integrates the serial and continuum mechanisms. As shown in Fig. 11C, this multitasking robot comprises a head and a neck [115]. The neck is designed based on the continuum mechanism, which can form an “S” shape and increase the workspace and flexibility of the robot. The head contains 2 discrete joints to enhance the robot's distal dexterity and motion accuracy. In addition, rigid link joints are inserted between continuum segments, as shown in Fig. 11D [83]. The compliant and flexible continuum segments provide inherent safe contact with surroundings. Moreover, the rigid link joints compensate for the nonlinear behavior of continuum segments and extend the workspace. Robots with the hybrid continuum-rigid structure feature trade-offs with precision and workspace compared to continuum robots.

Recently, a novel bending mechanism, referred to as the parallel continuum mechanism, was proposed and investigated [116] (see Fig. 11E). Compliant legs, usually super elastic tubes, were used to replace the rigid links of conventional parallel robots. A robot with this hybrid mechanism can retain some advantages, such as precision, reliability, and strength, of rigid-link parallel robots while inheriting some of the compactness and compliance of continuum robots.

## Variable Stiffness Mechanism

Safe access to the target surgical area through indirect, long, and tortuous intrabody routes is one of the specific technical challenges of TORS. To ensure adequate adaptability, conformability, and safety, surgical robots must have high flexibility, which can be achieved by reducing stiffness [117]. Nevertheless, lower stiffness leads to limited load capacity and reduced motion accuracy, hindering delicate and payload operations. Consequently, it is important for surgical robots to modulate their stiffness to meet the requirements presented by TORS in terms of safety, accuracy, and load capacity. This section has an overview of the variable stiffness (VS) mechanisms employed in the design of surgical robots, which can be divided into 3 groups on the basis of their working principles: phase-transition-based VS mechanism, jamming-based VS mechanism, and structure-based VS mechanism.

### Phase-transition-based VS mechanism

Mechanisms that take advantage of the mechanical properties of various phase-change materials at different states to achieve controllable stiffness are widely adopted in surgical robots.

**Fig. 12.**
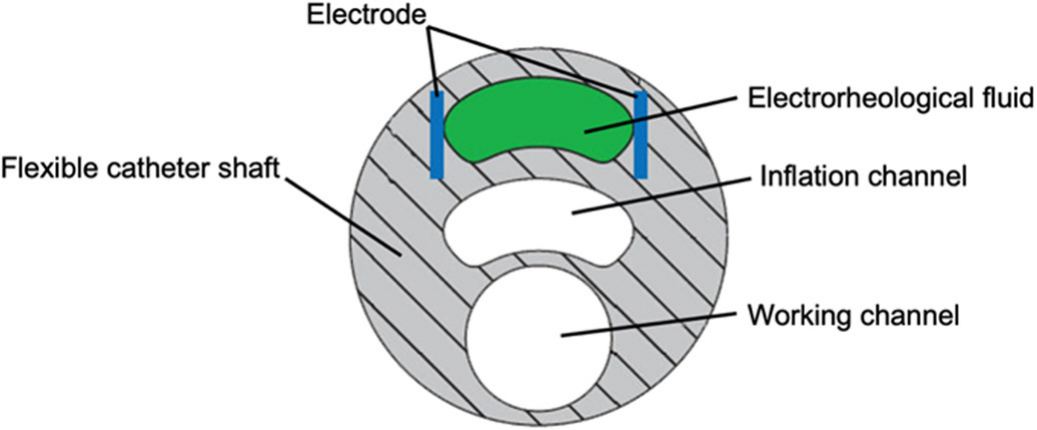
Surgical cross-sectional view of a balloon catheter with VS using electrorheological fluid [118].

Electrorheological fluid can transit between liquid and quasi-solid states by exposing it to the electric field, which was employed in designing a VS catheter [118]. As shown in Fig. 12, the electrorheological fluid is injected into the specific channel, with which the strength of the developed catheter can be increased from 0 to 5 kPa. However, it is far less than the clinical requirement. Moreover, high voltage and current are required to modulate the stiffness, which would endanger the patient.

**Fig. 13.**
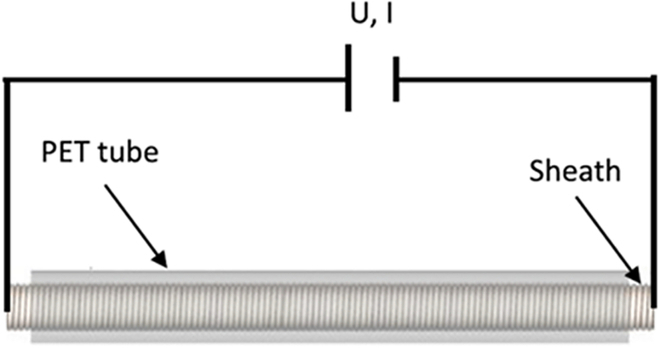
The working principle of variable-stiffness polyethylene terephthalate (PET) tube [120].

Thermoplastic materials can tune the load capacity of surgical robots. Polyurethane-based shape memory polymer was adopted in designing a catheter with VS, which obtains a high stiffness-changing ratio at the cost of long activation, cool time, and high transition temperature [119]. Le et al. [120] use polyethylene terephthalate (PET) tubes to achieve greatly adjusted stiffness and solve the above problems. As shown in Fig. 13, a flexible stainless-steel sheath was embedded to generate heat by applying current, reducing the activation time to less than 10 s. The inner channel of the sheath was utilized for active air cooling, with which the cooling time can be decreased from 100.3 to 11.9 s. In addition, aerogel was applied to keep the temperature of the outer face of the PET tube below 41°.

**Fig. 14.**
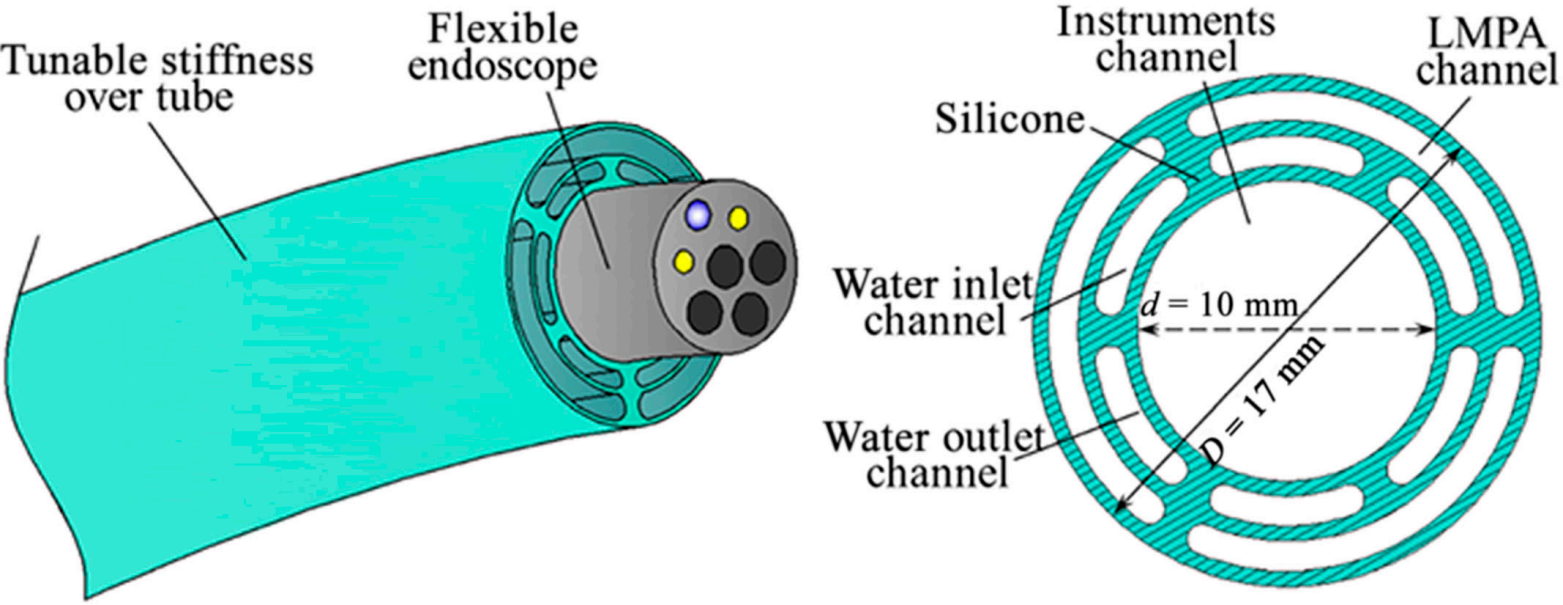
Illustration of VS over tube using low-melting-point alloy (LMPA) [121].

Low-melting-point alloys (LMPAs) are emerging solutions to stiffness adjustment for surgical robots, which can transit between the solid and liquid states at melting points close to the body temperature. Flexible surgical robots that use LMPAs to modulate the stiffness have a similar structure, as shown in Fig. 14. A variety of LMPAs, such as Cerrow 117 [121,122], Field’s metal [123], the liquid metal that is a mixture of indium, gallium, and stannum [124], and phase-change alloy [125], are in between tube gaps around the flexible backbone of the surgical robots. LMPAs can achieve faster phase change than thermoplastic materials due to their higher thermal conductivity [122]. However, even with active water or air circulation systems, the cooling time is still taking time. Besides, LMPAs contain toxic elements and would be a security risk to patients. Additional sealing measures should be taken to prevent leakage, increasing the structure's complexity and size.

### Jamming-based VS mechanism

**Fig. 15.**
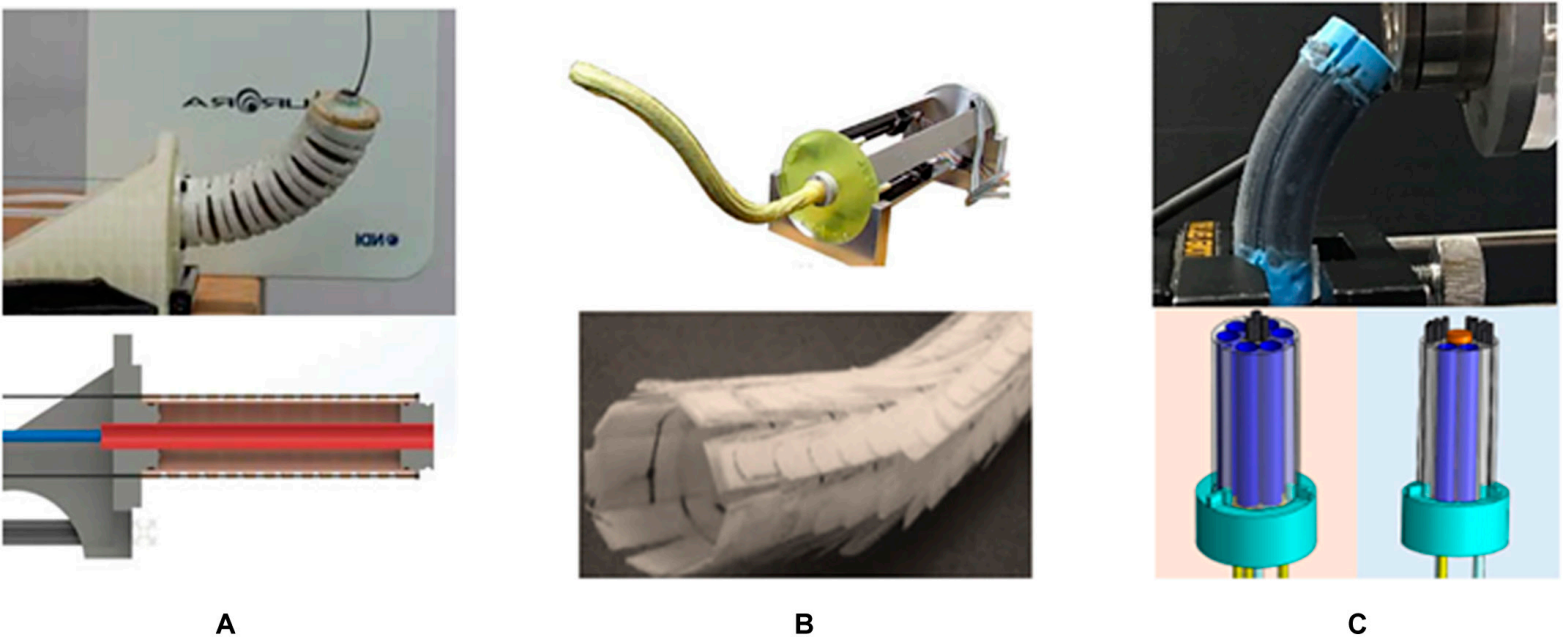
Surgical robots with VS using the jamming-based mechanism. (A) Granular jamming-based manipulator [126]. (B) Layer-jamming-based manipulator [128]. (C) Fiber-jamming-based manipulator [129].

Jamming-based VS mechanisms represent a group of solutions that achieve the transition between the rigid and flexible states by modifying the interactions between internal elements. As shown in Fig. 15, a variety of elements, including granules [126,127], layers [128], and fibers [129,130], have been employed to obtain the VS for surgical robots, which work on a similar principle. In the flexible state, elements can move freely within a small stiffness of the surgical robot. Once these elements are squeezed under external forces, frictions are greatly increased, leading to increased stiffness. As a result, the surgical robot can get locked in a given configuration. The most common way to exert the compression force is to generate the pressure difference using vacuum bumps, with which the external pressure can be much higher than the inside.

With jamming-based VS mechanisms, fast state transition can be achieved. Nevertheless, a high variation ratio of stiffness requires a substantial volume of elements and high-pressure difference, leading to a bulky structure design.

### Structure-based VS mechanism

Aside from relying on phase-change materials and jamming-based mechanisms, VS of surgical robots can also be obtained on the basis of structural characteristics.

**Fig. 16.**
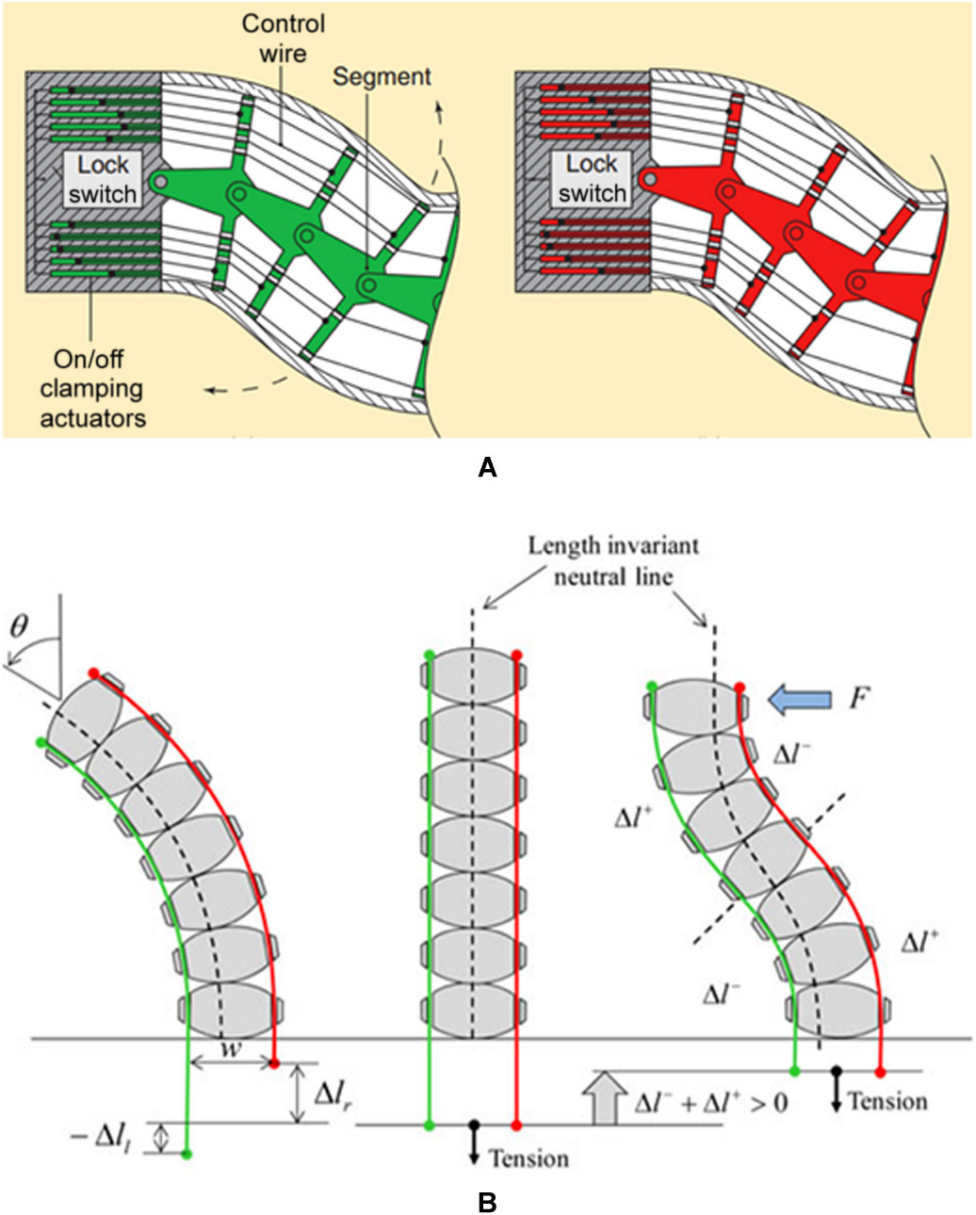
VS manipulators using tensioned cables. (A) Details of the NeoGuide^TM^ locking structure [117]. (B) Hyper-redundant with a variable neutral-line mechanism [177].

Cable-driven continuum mechanisms are widely employed in surgical robots, which can be investigated as a medium to change their stiffness. One typical example is the NeoGuide^TM^ Endoscopic Guide [116]. As shown in Fig. 16A, all cables are allowed to move freely when flexibility is required, which are simultaneously tensioned to make the distance between 2 adjacent segments unchanged to achieve shape locking. Since cable tensions can be instantly changed, the surgical robot can realize a quick switch between the flexible and stiff states. Nevertheless, a large number of cables and durable links are required to achieve the high stiffness-changing ratio, which hinders the scale down of the size. Other designs use tensioned cables to increase the friction between segments to enhance the rigidity of the structure, as shown in Fig. 16B. Although this design can use fewer cables to obtain the same stiffness changing the ratio, a complex control system or additional stiffness controlling mechanism is required to eliminate the coupling problem between the varying stiffness and positioning, which lead to the increased complexity in the robot design.

**Fig. 17.**
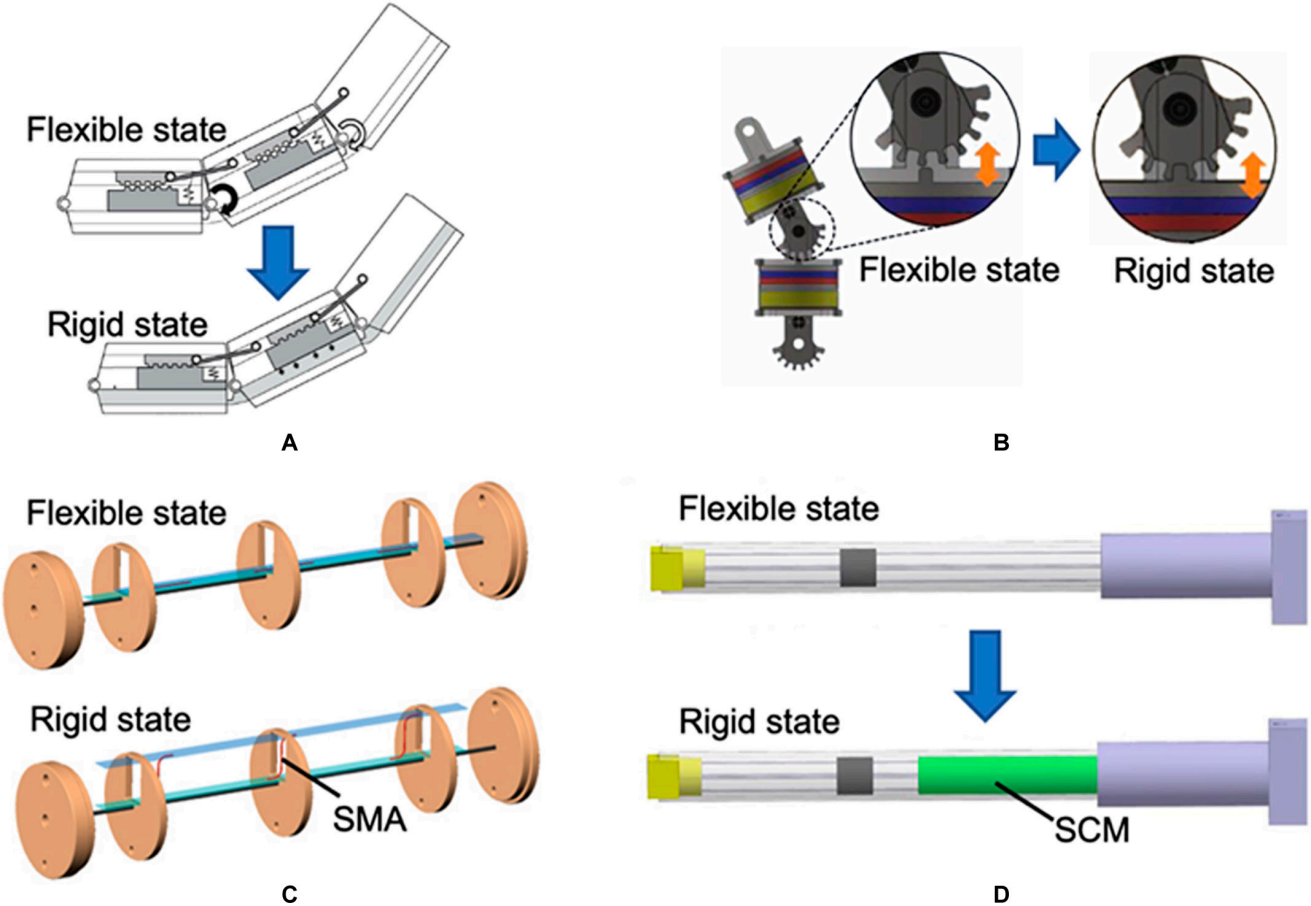
Structure-based VS mechanisms. (A) A slider-linkage-based locking mechanism [131]. (B) Shape-locking mechanism using a mechanical latch with electromagnetic force [132]. (C) Shape memory alloy (SMA)-based VS mechanism [133]. (D) Stiffness variation by actuation of stiffness-changing member (SCM) [134].

In addition to increasing cable tensions, the flexible surgical robot joints can be mechanically locked to achieve the VS. Figure 17A shows that a slider-linkage-based locking mechanism is developed to design an outer sheath model for endoscopic surgery [131]. When the air channel is empty, the slider can move freely to push the link to generate the bending motion. Once the air channel is filled with compressed air, the stopper is pushed to mesh the gear tooth of the slider to stop its movement. Consequently, this outer sheath model can be changed from a flexible state to a rigid state. The design depicted in Fig. 17B employs a similar design concept [132]. The electromagnetic force pushes the pawl to engage with the hook to stop the joint's rotation. With the above mechanisms, quick and high-strength shape locking is achievable. However, disadvantages, such as a limited number of locking positions, lack of continuously controllable stiffness, and complex structures, reduce the dexterity and maneuverability of the developed surgical robots.

As shown in Fig. 17C, Cao et al. [133] use the proactive deformation of shape memory alloy (SMA) wires to change the stiffness of a flexible manipulator for MIS. By inputting current, SMA wires transform into their memory shape, “S”, as depicted in the figure. The increased stiffness benefits from the change in the structure and the phase transition of the SMA. However, the phase transformation of SMA is accompanied by a change in temperature, which may cause additional harm to patients. Moreover, the long cooling times and low response frequency of SMA hinder the clinical application of the designed manipulator.

Different from the above mechanisms, a rigid stiffness-changing member (SCM) was introduced into the design of a continuum robot for neurosurgery, which can be manually moved to change the effective length of the continuum robot (see Fig. 17D) [134]. Shorter effective length results in a larger stiffness of the robot. The introduction of the SCM has little effect on the complexity and size of the structure. Additionally, the stiffness of the robot can be continuously tuned. However, the stiffness-changing ratio is smaller than the methods above. A motorized actuation system controls the SCM to make the stiffness adjustment more convenient and accurate.

## Triangulation Mechanism

The principle of triangulation is often described as the capacity of the surgical robot to obtain enough workspace and create adequate traction and counter traction for various operations, including the visualization, retraction, dissection, and suturing, with independently controllable manipulators [65,135,136]. Accordingly, several studies have revealed that triangulation is supposed to be a key element in the success of intraluminal procedures [137–141]. The triangulation can be easily achieved in a multiport extraluminal procedure by prearranged trocars, which becomes much more complex and difficult in MIS intraluminal procedures because multiple instruments are required to converge through the single incision or nature orifice [142]. This section overviews structures and mechanisms adopted in developing surgical robots to enable triangulation in deep and narrow surgical sites.

### Passive triangulation

SRSs for the MIS intraluminal procedure usually have a shaft whose distal ends serve as the base or provide working channels to manipulators equipped with various instruments or the vision unit. In many systems, the arrangement of the working channels at the distal end is preset and unchangeable. The triangulation is accomplished through the motion of manipulators, referred to as passive triangulation [142].

**Fig. 18.**
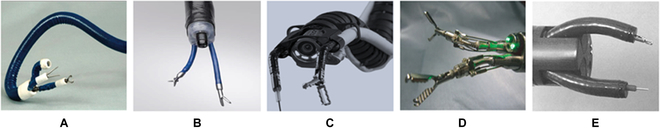
SRSs contain single bending segments to accompany the passive triangulation. (A) Cobra [136]. (B) Direct drive endoscopic system [178]. (C) Flex surgical system [52]. (D) A scorpion-shaped endoscopic surgical robot [179]. (E) A flexible endoscopic surgical system [180].

The simplest and most common configuration of passive triangulation is that manipulators are parallelly placed, each containing a single bending segment. Figure 18 shows some typical examples based on this configuration. Although bimanual operations are theoretically possible with this configuration, the workspace and the distal dexterity of these manipulators are subject to the dimension of the robot, leading to the limited application scope.

**Fig. 19.**
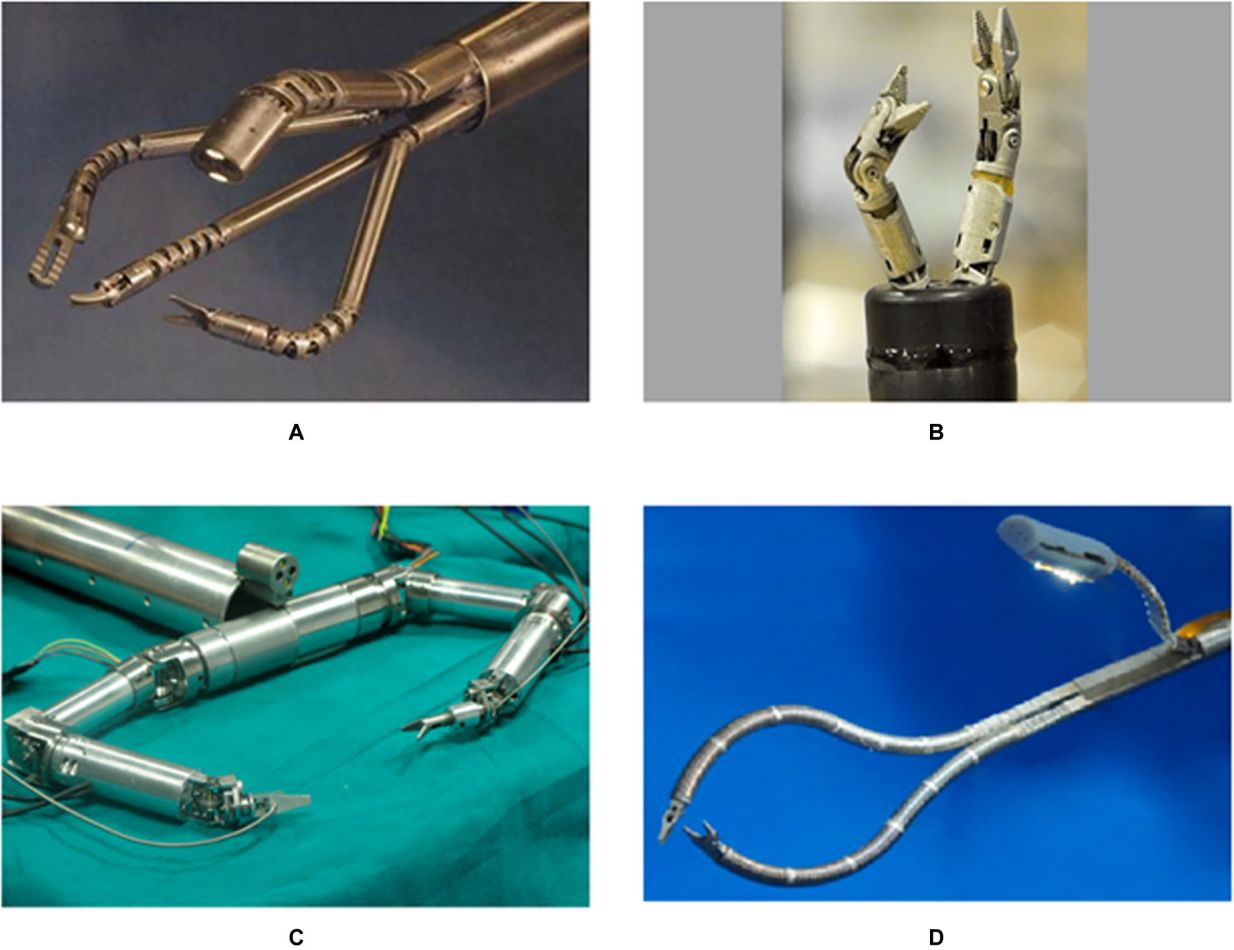
SRSs that have multiple joints or bending segments provide passive triangulation. (A) da Vinci SP system [143]. (B) EndoMaster [147]. (C) SPRINT [148]. (D) Shanghai Jiao Tong University unfoldable robotic system [150].

To obtain a larger workspace and improved distal dexterity, manipulators are supposed to engage tissues by deflections, which can be achieved using the following configurations. Figure 19A illustrates the mechanical structure of the da Vinci SP surgical robot, which contains an additional joint to generate the outward deflection and a bending section to complete the inward motion [143]. This configuration has been widely adopted in developing robots for MIS single-port surgery [125,144–146]. The EndoMaster robot-assisted surgical system [147] and the Single-Port lapaRoscopy bimaNual roboT (SPRINT) SRS [148] are composed of several pin joints to enable triangulation, as shown in Fig. 19B and C, with the proximal pin joint controlled to generate the deflection motion and the left pin joints steered to produce the inward motion. For manipulators that are designed on the basis of the continuum mechanism, the triangulation can be accomplished with multisegmented structures with the proximal continuum segment deflecting outward and the distal continuum segment bending inward to form an “S” shape [98,149,150] (see Fig. 19D). Although the increased workspace, flexibility, and distal dexterity can be achieved without increasing the size of the robots, these systems suffer from complex structures and coupling effects between joints or segments.

### Deployable mechanism

In addition to the passive triangulation, several mechanisms that allow the working channel of the manipulator to be radially deployed to enable the triangulation have been proposed and implemented in some surgical robot designs, which are referred to as the deployable mechanism.

**Fig. 20.**
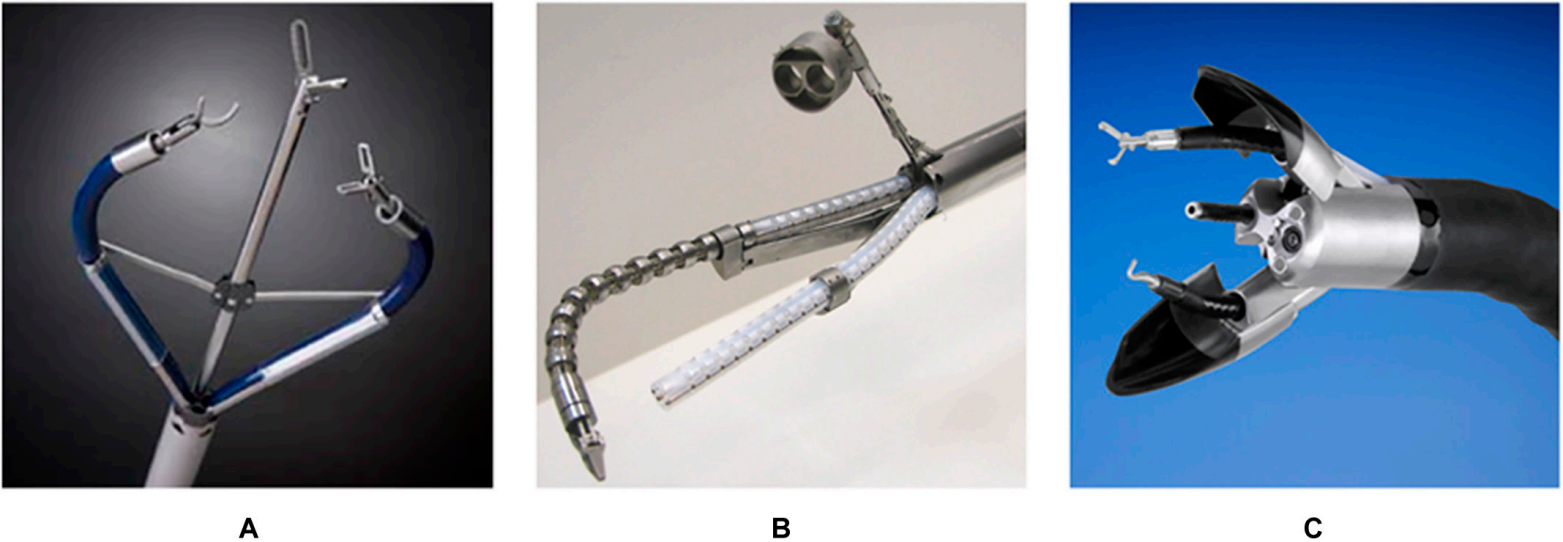
SRSs with deployable mechanisms. (A) TransEnterix SPIDER [151]. (B) Insertable robotic effector platform [152]. (C) Single access and Transluminal Robotic Assistant for Surgeons (STARAS) [153].

TransEnterix Single-Port Instrument Delivery Extended Research (SPIDER) is a manual surgical system targeting SP surgery, the distal part comprising a retractable sheath and 4 working channels [151]. After being inserted through the incision and reaching the surgical site, the sheath is pulled back, and the 2 active channels are driven to a deployed configuration by a slider-crank mechanism to provide the triangulation, as shown in Fig. 20A. In this system, the distance between 2 active channels can be adjusted within a large range, contributing to a larger workspace, improved distal dexterity, and broader application scope. Nevertheless, this system's motion accuracy and maneuverability are subject to manual operation.

Insertable robotic effector platform is a self-deployable SRS in a working configuration in a single-port surgery [152]. As shown in Fig. 20B, a parallelogram mechanism is adopted to improve the triangulation of the surgical robot, with which the base of the snake-like manipulators can be controlled to move outward according to the requirements of the surgical operation. In addition to the increased workspace and distal dexterity, this deployable mechanism affords improved stability and maneuverability compared to the SPIDER system. Nevertheless, a number of delicate parts are required in the parallelogram mechanism, which leads to a high manufacturing cost. Furthermore, the reliability of this mechanism should be investigated with more experiments.

As illustrated in Fig. 20C, STARAS is the modification of the manual Anubiscope^TM^ for the intraluminal procedure [153]. The distal part of this robot contains 2 streamlined shells that contain the working channels for manipulators, which are closed to facilitate the advancement of the robot in narrow environments during the insertion phase. Once the target surgical site is reached, shells are unfolded to make the working channels deviate from the original direction, which can provide triangulation during the procedure. Two pairs of antagonistic cables can independently control the shells. Thus, this deployable mechanism is suitable for robots with a flexible shaft and contributes to a simple structure. However, the motion range of the shell is limited, the maximum deflection angle of which is only about 60°.

## Comparative Discussions and Future Perspectives

Table 2 summarizes typical structure sketches and several performance parameters of different distal dexterity mechanisms, including the DOF, load capacity, and outer diameter. In addition, the features of these mechanisms are described in Table 3. As evident in both tables, the serial mechanism and PM usually have high stiffness and can achieve relatively higher load capacity, distal dexterity, and motion accuracy. However, reduced flexibility and compliance, as well as the difficulty of minimization, make them only applicable to some extraluminal procedures. Continuum mechanisms have infinite DOFs and intrinsic compliance to ensure better adaptability and safety in narrow and complex environments, which have been widely adopted in developing surgical robots for both extraluminal and intraluminal procedures. However, limited dexterity, load capacity, and accuracy hinder the clinical application of these robots.

**Table 2.** Typical structures and performance parameters of distal dexterity mechanisms

Mechanisms	Typical structure sketch	DOF	Payload	Outer diameter	Applicable surgery
Serial mechanism	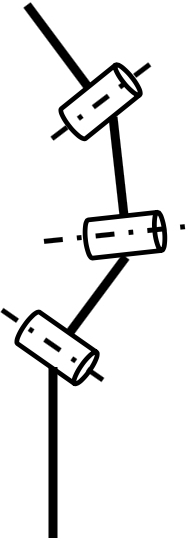	2–6	≥3 N	3–18 mm	Extraluminal procedures
Parallel mechanism	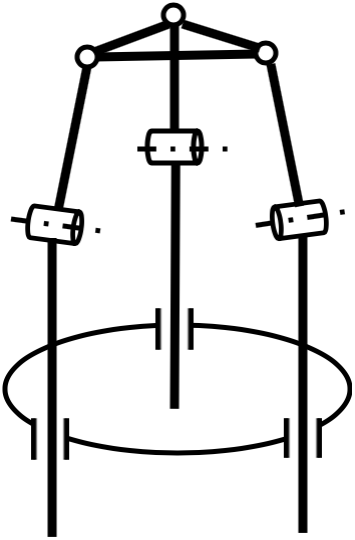	3–6	3–10 N	6–10 mm	Extraluminal procedures
Continuum mechanism	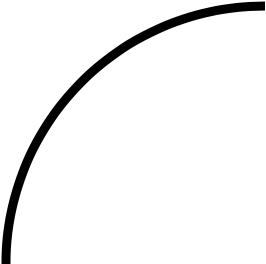	Infinite	0.5–2 N	1.07–32 mm	Extraluminal and intraluminal procedures
Hybrid mechanism	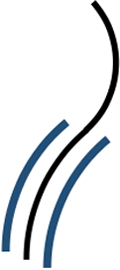	Infinite	5 N	12 mm	Extraluminal and intraluminal procedures
	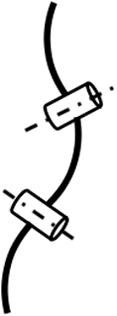	Infinite	-	13–16 mm	Extraluminal and intraluminal procedures
	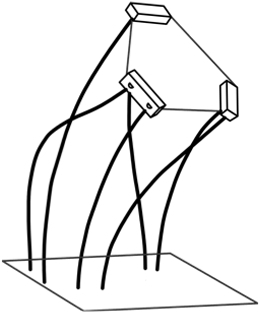	6	5 N	12 mm	Extraluminaland intraluminal procedures

DOF, degree of freedom

**Table 3.** Features of different distal dexterity mechanisms

	Serial robots	Continuum robots	Parallel robots	Hybrid mechanisms
Workspace	**+++**	**+++**	**+**	**+++++**
Flexibility	**+**	**+++++**	**+**	**+++++**
Dexterity	**+++**	**+**	**+++++**	**+++++**
Compliance	**+**	**+++++**	**+**	**+++++**
Load capacity	**+++++**	**+**	**+++++**	**+++**
Accuracy	**+++++**	**+**	**+++++**	**+++++**
Scaling down	**+**	**+++++**	**+**	**+++**

**+** represents relatively poor performance. **+++** represents normal performance. **+++++** represents relatively good performance.

Hybrid mechanisms can draw on individual mechanism strengths to achieve tremendously improved performance over other mechanisms in terms of workspace, flexibility, dexterity, compliance, and accuracy. Consequently, a novel hybrid

mechanism that is similar to the continuum PM and can satisfy requirements presented by the TORS is proposed in [154]. It is further employed in the development of a surgical robot with VS that is targeted at the oropharyngeal cancers in [155] and is combined with a continuum mechanism that can cover the entire UAT in [156].

VS of the surgical robot can be achieved on the basis of different principles, which have respective merits and demerits. The advantages of the phase-transition-based approaches include the large stiffness changing the ratio, and simple structure. Jamming-based mechanisms can achieve a quick switch between the flexible and stiff modes. Nevertheless, it is difficult to scale down the size of robots based on this mechanism while maintaining the same stiffness-changing ratio. Structure-based mechanisms are capable of fast and continuous stiffness adjustment and easy minimization, which is more suitable for medical applications. Thus, the surgical robot utilizing the structural features of the flexible PM can effectively achieve the VS. Drawbacks of current designs, such as small stiffness changing the ratio, complicated controllers, and high production cost, can be overcome by the mechanical design.

Most SRSs adopt passive triangulation configurations to enhance the ability of the surgical robot to complete surgical operations. In these designs, manipulators are required to have more controllable joints or bending segments, which lead to reduced motion accuracy, load capacity, and stability. Deployable mechanisms can effectively alleviate these problems, which can adjust the working channels or the base of manipulators in a wider range and offer improved stability and load capacity.

New robotic technologies can contribute to significant improvements in the quality of life and survival for patients with HNCs [157] and also play an important role in promoting the technical advancement of other intraluminal procedures, e.g., transanal colorectal microsurgery [158,159], transurethral bladder procedures [160,161], transvaginal abdominal surgery [162,163], and transesophageal thoracic surgery [164,165].

New robotic technologies shall address challenges imposed by the TORS and develop surgical robots with improved performance to overcome the limitations of current designs and explore the full potential of TORS. New mechanisms, particularly flexible PMs that can adopt the flexible deformation of super elastic materials instead of rigid links and complex components as adopted in the traditional PM, are promising to generate flexible and dexterous motions aiming at addressing challenges presented by TORS in terms of the size, workspace, flexibility, dexterity, load capacity, and safety.

## Conclusion

This paper reviewed mechanisms related to distal dexterity, VS, and triangulation in developing SRSs. We discuss the TORS background, significance, and technical challenges. Finally, conclusions about the work and future perspectives of the flexible surgical robots for TORS are summarized to provide references for future robot designs.

New SRS developments shall resolve the contradiction between flexibility, motion accuracy, and load capacity. Future SRS contains manipulators with VS that can possess sufficient compliance to ensure adequate safety while keeping an acceptable stiffness level for manipulation with adequate precision in the confined surgical site, which can be used in managing cancers in the oropharynx.

## Data Availability

No additional datasets were generated or analyzed during the current study.
